# *ℓ*_*p*_-Norm Multikernel Learning Approach for Stock Market Price Forecasting

**DOI:** 10.1155/2012/601296

**Published:** 2012-12-29

**Authors:** Xigao Shao, Kun Wu, Bifeng Liao

**Affiliations:** ^1^School of Mathematics and Statistics, Central South University, Changsha, Hunan 410075, China; ^2^Wengjing College, Yantai University, Yantai, Shandong 264005, China; ^3^School of Mathematics and Information Science, Yantai University, Yantai, Shandong 264005, China

## Abstract

Linear multiple kernel learning model has been used for predicting financial time series. However, *ℓ*_1_-norm multiple support vector regression is rarely observed to outperform trivial baselines in practical applications. To allow for robust kernel mixtures that generalize well, we adopt *ℓ*_*p*_-norm multiple kernel support vector regression (1 ≤ *p* < *∞*) as a stock price prediction model. The optimization problem is decomposed into smaller subproblems, and the interleaved optimization strategy is employed to solve the regression model. The model is evaluated on forecasting the daily stock closing prices of Shanghai Stock Index in China. Experimental results show that our proposed model performs better than *ℓ*_1_-norm multiple support vector regression model.

## 1. Introduction

Forecasting the future values of financial time series is an appealing yet difficult activity in the modern business world. As explained by Deboeck and Yaser [1, 2], the financial time series are inherently noisy, nonstationary, and deterministically chaotic. In the past, many methods were proposed for tackling this kind of problem. For instance, the linear models for forecasting the future values of stock prices include the autoregressive (AR) model [3], the autoregressive moving average (ARMA) model [4], and the autoregressive integrated moving average (ARIMA) model [4]. Over the last decade, nonlinear approaches have received increasing attention in financial time series prediction and have been proposed for a satisfactory answer to the problem. For example, Yao and Tan [5] used time series data and technical indicators as the input of neural networks to increase the forecast accuracy of exchange rates; Cao and Tay [6, 7] applied support vector machine (SVM) in financial forecasting and compared it with the multilayer back-propagation (BP) neural network and the regularized radial basis function (RBF) neural network; Qi and Wu [8] proposed a multilayer feed-forward network to forecast exchange rates; Pai and Lin [9] invested a hybrid ARIMA and support vector machines model in stock price forecasting; Pai et al. [10] presented a hybrid SVM model to exploit the unique strength of the linear and nonlinear SVM models in forecasting exchange rate; Kwon and Moon [11] proposed a hybrid neurogenetic system for stock trading; Hung and Hong [12] presented an improved ant colony optimization algorithm in a support vector regression (SVR) model, called SVRCACO, for selecting suitable parameters in exchange rate forecasting; Jiang and He [13] introduced local grey SVR (LG-SVR) integrated grey relational grade with local SVR for financial times eries forecasting; and so on.

In comparison with the previous models, SVR with a single kernel function can exhibit better prediction accuracy because it conceives the structural risk minimization principle which considers both the training error and the capacity of the regression model [14, 15]. However, the researchers have to determine in advance the type of kernel function and the associated kernel hyper parameters for SVR. Unsuitably chosen kernel functions or hyper parameter settings may lead to significantly poor performance [16, 17].

In recent years there has a lot of interest in designing principled regression algorithms over multiple cues, based on the intuitive notion that using more features should lead to better performance and decreasing the generalization error. When the right choice of features is unknown, learning linear combinations of multiple kernels is an appealing strategy. The approach with a optimization process is called multiple kernel learning (MKL). A first step towards a more realistic model of MKL was achieved by Lanckriet et al. [18], who showed that, given a candidate set of kernels, it is computationally feasible to simultaneously learn a support vector machine and a linear kernel combination at the same time. In MKL we need to solve a joint optimization problem while also learning the optimal weights for combing the kernels. Several practitioners have adopted the linear multiple kernels to deal with the practical problems. For example, Rakotomamonjy et al. [19] addressed the MKL problem through a weighted 2-norm regularization formulation and proposed an algorithm, named Simple MKL, for solving this MKL problem. Bach [20] proposed the asymptotic model consistency of the group Lasso. Zhang and Shen [21] presented multimodal multitask learning algorithm for joint prediction of multiple regression and classification variables in Alzheimer's disease. Especially, Chi-Yuan Yeh and his coworkers [22] developed a two-stage MKL algorithm by incorporating sequential minimal optimization and the gradient projection method. The new method [22] performed better than previous ones for forecasting the financial time series. Previous approaches to multiple kernel learning (MKL) have promoted sparse kernel combinations to support interpretability and scalability. Unfortunately, sparsity at the kernel level may harm the generalization performance of the learner, therefore *ℓ*_1_-norm MKL is rarely observed to outperform trivial baselines in practical applications [23]. To allow for robust kernel mixtures that generalize well, the researchers extend *ℓ*_1_-norm MKL to arbitrary norms, that is, *ℓ*_*p*_-norm MKL (1 ≤ *p* < *∞*). For example, Marius Kloft et al. developed two efficient interleaved strategies for *ℓ*_*p*_-norm MKL and showed that it can achieve better accuracy than *ℓ*_1_-norm MKL for real-world problems [23]; Francesco Orabona et al. presented a MKL optimization algorithm based on stochastic gradient descent for *ℓ*_*p*_-norm MKL, which possessed a faster convergence rate as the number of kernels grows [24].

In this paper, a multiple kernel learning framework is established for learning and predicting the stock prices. We present a regression model for the future values of stock prices, that is, *ℓ*_*p*_-norm multiple kernel support vector regression (*ℓ*_*p*_-norm MK-SVR), where 1 ≤ *p* < *∞*. We decompose the optimization problem into smaller subproblem and adopt the interleaved optimization strategy to solve the regression model. Our experimental results show that *ℓ*_*p*_-norm MK-SVR performs a better performance.

The rest of this paper is arranged as follows.  Section 2 details the processing of the *ℓ*_*p*_-norm MK-SVR model construction and describes the algorithm for our regression model. Experimental results are presented in Section 3.  Section 4 concludes the paper and provides some future research directions.

## 2. Forecasting Methodology

### 2.1. *ℓ*_*p*_-Norm Multiple Kernel Support Vector Regression

In this section, the idea of *ℓ*_*p*_-norm multiple kernel support vector regression (*ℓ*_*p*_-norm MK-SVR) is introduced formally.

Let {**x**_*i*_, *y*_*i*_}_*i*=1_^*N*^, where **x**_*i*_ ∈ **R** ^*n*^ and *y*_*i*_ ∈ **R**, be the training set. Each *y*_*i*_ is the desired output value for the input vector **x**_*i*_. Consider a function *ϕ*(**x**_*i*_) : **R** ^*n*^ → **H** that maps the samples into a high, possibly infinite, dimensional space. A regression model is learned from the previous and used to predict the target values of unseen input vectors. SVR is a nonlinear kernel-based regression method which tries to locate a regression hyperplane with small risk in high-dimensional feature space [14]. Considering the soft margin formulation, the objective function and constraints for SVR should be solved, as follows:

(1) minw~,b λ2〈w~,w~〉+1N∑i=1l(ξi+ξ^i)s.t. (〈w~,ϕ(xi)〉+b)−yi≤ε+ξi,  yi−(〈w~,ϕ(xi)〉+b)≤ε+ξ^i,  ξ,ξ^i≥0, i=1,2,…,N.

SVR model usually uses a single mapping function *ϕ* and hence a single kernel function *K*. Although the SVR model has good function approximation and generalization capabilities, it is not fit for dealing with a data-set which has a locally varying distribution. For resolving this problem, we can construct a MK-SVR model. Combining multiple kernels instead of using a single one, *ℓ*_*p*_-norm MK-SVR model can catch up the varying distribution very well. Therefore we can use the composite feature map *ϕ* which has a block structure:

(2) $$\begin{array}{c} \phi \left(x\right)=\left[\sqrt{d_{1}}\phi_{1}\left(x\right)\times \sqrt{d_{2}}\phi_{2}\left(x\right)\times \cdots \times \sqrt{d_{M}}\phi_{M}\left(x\right)\right] \end{array}$$

to map the input space to the feature space, where *d*_1_, *d*_2_,…, *d*_*M*_ are weights of component functions. Given a set of base kernels *K*_*k*_ which correspond the previous feature maps {*ϕ*_*k*_}(*k* = 1,2,…, *M*), linear MK-SVR aims to learn a linear combination of the base kernels as *K* = ∑_*k*_ *d*_*k*_ *K*_*k*_. In learning with MK-SVR we aim at minimizing the loss on the training data with respect to the optimal kernel mixture ∑_*k*_ *d*_*k*_ *K*_*k*_ in addition to regularizing **d** to avoid overfitting. The primal can therefore be formulated as

(3) minw~,b  λ2(∑k||w~k||2)2+1N∑i=1l(ξi+ξ^i)+μ~Ω~[d]s.t.  (〈w~,K(xi)〉+b)−yi≤ε+ξi,yi−(〈w~,K(xi)〉+b)≤ε+ξ^i, μ~>0,d1,d2,…,dM≥0,ξ,ξ^i≥0, i=1,2,…,N.

Previous research to MK-SVR employs the regularizer of the form $\tilde{\Omega }[d]=||d{||}_{1}(d=(d_{1},d_{2},\ldots ,d_{M}))$ which can promote sparse kernel mixtures. However, sparsity is not always desirable, since the information carried in the zero-weighted kernels is lost. Therefore we propose to use nonsparse and thus more robust kernel mixtures by employing an *ℓ*_*p*_-norm constraint with *p* > 1, that is, $\tilde{\Omega }[d]=||d{||}_{p}^{2}$, and ||**d**||_*p*_ = (∑_*k*_ *d*_*k*_^*p*^)^1/*p*^, 1 < *p* < *∞*. In (3), let $\sqrt{d_{k}}{\tilde{w}}_{k}=w_{k}$, *C* = 1/*nλ*, $\tilde{\mu }=\mu \lambda$, and the first equation be divided with *λ*, then the following *ℓ*_*p*_-norm MK-SVR is obtained:

(4) minw,b  12∑k||wk||22dk+C∑i=1l(ξi+ξ^i)+μ||d||p2s.t. (〈w,K(xi)〉+b)−yi≤ε+ξi,yi−(〈w,K(xi)〉+b)≤ε+ξ^i,μ>0,d1,d2,…,dM≥0,ξ,ξ^i≥0, i=1,2,…,N.

An alternative approach previous equations has been considered by studiers. For example, Zien and Ong [25] upperbound the value of the regularizer ||**d**||_1_ and incorporate the regularizer as an additional constraint into the optimization problem. According to this thought, *ℓ*_*p*_-norm MK-SVR model (4) can be transformed into the following form:

(5) minw,b 12∑k||wk||22dk+C∑i=1l(ξi+ξ^i)s.t. (〈w,K(xi)〉+b)−yi≤ε+ξi,yi−(〈w,K(xi)〉+b)≤ε+ξ^i,||d||p2≤1,d1,d2,…,dM≥0,ξ,ξ^i≥0, i=1,2,…,N.

It can be shown (see the Appendix for details) that the dual of (5) is

(6) maxα^,α  (yT(α^−α)−ε(α^+α))  −||(12dk∑i=1N∑j=1N(α^i−αi)(α^j−αj)Kk(xi,xj))k=1M||p∗s.t. 1T(α^−α)=0,0≤α^,α≤C1,d1,d2,…,dM≥0,

where **y** = (*y*_1_, *y*_2_,…, *y*_*N*_)^*T*^, **ε** = (1,1,…, 1)^*T*^, **α** = (*α*_1_, *α*_2_,…, *α*_*N*_)^*T*^ ∈ **R** ^*N*^, $\hat{\alpha }={\left({\hat{\alpha }}_{1},{\hat{\alpha }}_{2},\ldots ,{\hat{\alpha }}_{N}\right)}^{T}\in R^{N}$, and p* = *p*/(*p* − 1) is the dual norm of *p*. Suppose the optimal ${\hat{\alpha }}_{i}^{*}$, *α*_i_*(*i* = 1,2,…, *N*) and *d*_1_*, *d*_2_*,…, *d*_M_* are found by solving (6), the regression hyperplane for *ℓ*_*p*_-norm MK-SVR model is given by

(7) $$\begin{array}{c} f^{*}\left(x\right)=\sum_{i=1}^{N}\left({\hat{\alpha }}_{i}^{*}-\alpha_{i}^{*}\right)K\left(x_{i},x\right)+b^{*}, \end{array}$$

where $b^{*}=y_{j}+\varepsilon -\sum_{i=1}^{N}\left({\hat{\alpha }}_{i}^{*}-\alpha_{i}^{*}\right)K(x_{i},x_{j})$ is obtained from any ${\hat{\alpha }}_{i}^{*}$ and *α*_i_*, with $0<{\hat{\alpha }}_{i}^{*},\alpha_{i}^{*}<C1$. In the following section, an efficient algorithm is proposed for solving the optimization problem (6).

### 2.2. An Optimistic Algorithm

**Figure 1:**
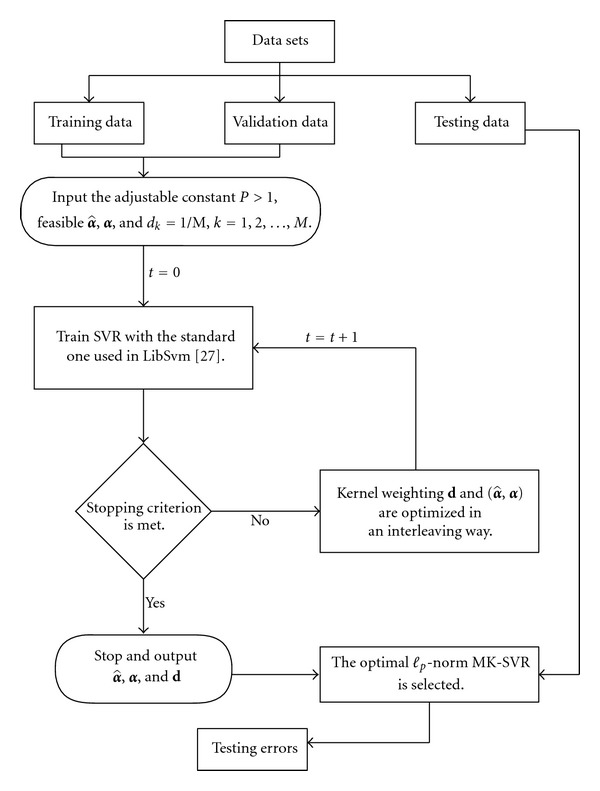
*ℓ*_*p*_-norm MK-SVR model learning algorithm (see [27]).

*ℓ*_*p*_-norm MK-SVR model (6) can be trained with several algorithms, for example, the Sequential Minimal Optimization algorithm [26] and multi-kernel learning with online-bath optimization [24]. In this paper, the interleaved optimization is used for the optimization scheme according to the idea of [23]. As a matter of fact, we can exploit the structure of *ℓ*_*p*_-norm MK-SVR cost function by alternating between optimizing the linear combination of the base kernels *K* = ∑_*k*_ *d*_*k*_ *K*_*k*_ and the remaining variables as $\hat{\alpha }$ and **α**. We can do so by setting up a two-stage optimization algorithm. The basic idea of the algorithm is to divide the optimization variables of *ℓ*_*p*_-norm MK-SVR problem (6) into two groups, $\left(\hat{\alpha },\alpha \right)$ on one hand and **d** = (*d*_1_, *d*_2_,…, *d*_*M*_) on the other. Our procedure will alternatingly operate on those two stages via a block coordinate descent algorithm. Therefore the optimization **d** will be carried out analytically and the $\left(\hat{\alpha },\alpha \right)$ will be computed in the dual. The two stages are iteratively performed until the specified stopping criterion is met, as shown in Figure 1.

In the first stage, the variables $\left(\hat{\alpha },\alpha \right)$ are kept fixed, that is, the $\left(\hat{\alpha },\alpha \right)$ are known. Then the optimal **d** in *ℓ*_*p*_-norm MK-SVR model (6) can be calculated analytically by the following process.

According to ((A.4)), let

(8) L=∑i=1Nyi(α^i−αi)−ε∑i=1N(α^i+αi)−  12∑k=1Mdk∑i=1N∑j=1N(α^i−αi)(α^j−αj)Kk(xi,xj)+β(12||d||p2−12)−γTd.

Set the *L*'s first partial derivatives with respect to *d*_*k*_, and let it be 0:

(9) ∂L∂dk=0⇒β(∑kdkp)2/p−1dkp−1=γk+12∑i=1N∑j=1N(α^i−αi)(α^j−αj)Kk(xi,xj)⇒β(∑kdkp)2/p=∑kdk(γk+12∑i=1N∑j=1N(α^i−αi)     ×(α^j−αj)Kk(xi,xj)).

In the optimal point *γ*_*k*_ = 0 holds, so the previous equation yields

(10) dk=12β(∑k∑i=1N∑j=1N(α^i−αi)(α^j−αj)Kk(xi,xj))(1/q)−(1/p)×(∑i=1N∑j=1N(α^i−αi)(α^j−αj)Kk(xi,xj))q/p,

where (1/*p*)+(1/*q*) = 1, and *k* = 1,2,…, *M*.

In the second stage, the following algorithm is used. We give a chunking-based training algorithm (Algorithm 1) via analytical update for *ℓ*_*p*_-Norm MK-SVR. Kernel weighting **d** and $\left(\hat{\alpha },\alpha \right)$ are optimized in an interleaving way. The basic idea of this algorithm is to divide the optimal problem into an inner subproblem and an outer subproblem. The algorithm alternates between solving the two subproblems until convergence.

In every iteration process, the inner subproblem ($\hat{\alpha }$ and **α** step) identifies the constraint that maximises (6) with fixing kernel weighting **d**. The outer subproblem (**d** step) is also called the restricted master problem. *d*_*k*_ is computed with the (10), *k* = 1,2,…, *M*.

The interleaved optimization algorithm is depicted in Algorithm 1, and the details of it are as follows.

#### 2.2.1. Initialization

Assume the original values of ${\hat{\alpha }}_{i}$ and *α*_*i*_ are 0, for  all *i* = 1,2,…, *N*, and the initial value of *d*_*k*_ is ${\sqrt{1/k}}^{p}$, for all *k* = 1,2,…, *M*, where *p* > 1 is a constant.

#### 2.2.2. Chunking and Carrying out with SVR

In the iteration process, the procedure is standard in chunking-based SVR solvers and is carried out by SVM^light^, where *Q* is chosen as described in [28]. We implement the greedy second-order working set selection strategy of [28]. Rather than compute the gradient repeatedly, we speed up variable selection by caching, separately for each kernel. The cache needs to be updated every time we change ${\hat{\alpha }}_{Q}$ and **α**_*Q*_ in the reduced variable optimisation. In Algorithm 1, (4) and (5) compute the objective values of SVR. Finally, the analytical value of **d** is carried out in (10).

#### 2.2.3. Stopping Criterion

When the duality gap falls below a prespecified threshold, that is, |1 − ((*L* − *S*)/(*L*_old_ − *S*_old_))|<*ε*, we terminate the algorithm and output $\hat{\alpha }$, **α**, **d**.

## 3. Experimental Results

 In this section, two experiments on a real financial time series have been carried out to assess the performance of *ℓ*_*p*_-norm MK-SVR. The motivation behind the two experiments are to compare the performance of our proposed method with that of other methods, that is, single kernel support vector regression (SKSVR) [29] and *ℓ*_1_-norm MK-SVR [22]. All calculations are performed with programs developed in MATLAB R2010a.

### 3.1. Experiment I

 Firstly, we compare the performance of *ℓ*_*p*_-norm MK-SVR with that of SKSVR. In this experiment, the daily stock closing prices of Shanghai Stock Index in China for the period of January 2003 to December 2007 are used, and the training/validating/testing data set is generated by a one-season moving-window testing approach. Following the way done in [29], three data sets, data1 to data3, are formed. For instance, data1 contains the daily stock closing prices from January 2003 to December 2006 are selected as the training data set, the daily stock closing prices from January 2007 to March 2007 are selected as the validating data set, the daily stock closing prices from April 2007 to June 2007 are selected as the testing data set. The corresponding time periods for data 1 to data 3 are listed in Table 1.

According to [29], we can derive training patterns (**x**_*t*_, *y*_*t*_) based on the original daily stock closing prices **P** = {*p*_1_,…, *p*_*t*_…} for SKSVR and *ℓ*_*p*_-norm MK-SVR. Let EMA_*n*_(*t*) = EMA_*n*_(*t* − 1) + *α* × (*p*_*t*_ − EMA_*n*_(*t* − 1)) be the *n*-day exponential moving average of the *t*th day, where *p*_*t*_ is the *t*th day daily stock closing prices and *α* = 2/(*n* + 1), then the output variable *y*_*t*_ can be defined as

(11) $$\begin{array}{c} y_{t}=\text{RD}{\text{P}}_{+5}\left(t\right)=\frac{\text{EM}{\text{A}}_{3}t-\text{EM}{\text{A}}_{3}\left(t-5\right)}{\text{EM}{\text{A}}_{3}\left(t-5\right)}\times 100. \end{array}$$

Let **x**_*t*_ = (*x*_*t*,1_, *x*_*t*,2_, *x*_*t*,3_, *x*_*t*,4_, *x*_*t*,5_) be the input vector and let RDP_−*n*_(*t*) = (100 × (*p*_*t*_ − *p*_*t*−*n*_))/*p*_*t*−*n*_ be the lagged relative difference in percentage of price (RDP). Moreover, We can obtain a transformed closing price $\text{E}\tilde{\text{W}}{\text{A}}_{n}(t)$ by subtracting a *n*-day EMA from the closing price, that is,

(12) $$\begin{array}{c} \text{E}\tilde{\text{W}}{\text{A}}_{n}\left(t\right)=p_{t}-\text{EW}{\text{A}}_{n}\left(t\right). \end{array}$$

Based on in the previously mentioned, the input variables can be defined as $x_{t,1}=\text{E}\tilde{\text{W}}{\text{A}}_{15}(t-5)$, $x_{t,2}=\text{R}\tilde{\text{D}}{\text{P}}_{-5}(t-5)$, $x_{t,3}=\text{R}\tilde{\text{D}}{\text{P}}_{-10}(t-5)$, $x_{t,4}=\text{R}\tilde{\text{D}}{\text{P}}_{-15}(t-5)$, and $x_{t,3}=\text{R}\tilde{\text{D}}{\text{P}}_{-20}(t-5)$. We adopt the root mean squared error (RMSE) for performance comparison, that is,

(13) $$\begin{array}{c} \text{RMSE}=\sqrt{\frac{1}{T}\sum_{t=1}^{T}{\left(y_{t}-{\hat{y}}_{t}\right)}^{2}}, \end{array}$$

where *y*_*t*_ and ${\hat{y}}_{t}$ are desired output and predicted output, respectively.

**Figure 2:**
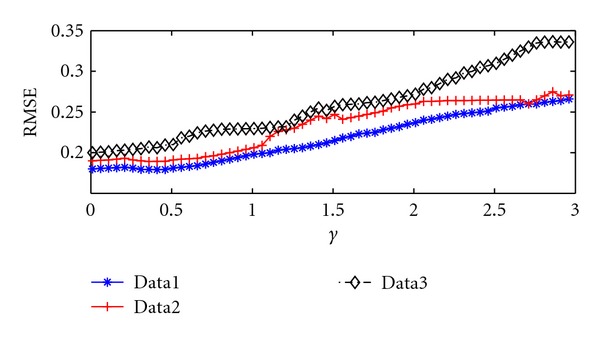
Forecasting performance of SKSVR with different hyperparameters.

There are three parameters that should be determined in advance for SKSVR,  that is, *C*, *ε*, and *γ* for using RBF kernel. The forecasting performance of SKSVR is examined with *C* = 1 and *ε* = 0.005. Because the forecasting performance obtained by SKSVR is effected by the parameter *γ*, we try with different settings of it from 0.01 to 3 with a stepping factor of 0.05.  Figure 2 shows the RMSE for performance on the three data sets by SKSVR. The figure shows that SKSVR requires different *γ* settings for different data sets to obtain the best performance. For example, the best performance for data 1 occurs when 0.35 ≤ *γ* ≤ 0.45. The best RMSE values obtained by SKSVR are listed in Table 2.

For *ℓ*_*p*_-norm MK-SVR training model, we adopt RBF kernel *K*(**x**, **x**_*k*_) = exp{−||**x**_*i*_ − **x**_*j*_||_2_^2^/*σ* ^2^}. A kernel combining 60 different RBF kernels is considered,  that is, 0.01 ≤ 1/*σ* ^2^ ≤ 3 with step 0.05. Hence, the kernel matrix is combined with a weighted sum of 60 kernel matrices, that is, $\tilde{K}=d_{1}K_{1}+d_{2}K_{2}+\cdots +d_{60}K_{60}$ where *d*_1_ denotes the kernel weight for the first kernel matrix with 1/*σ* ^2^ = 0.01 and *d*_2_ denotes the kernel weight for the second kernel matrix with 1/*σ* ^2^ = 0.06, and so on. For the three data sets, the RMSE values obtained by *ℓ*_*p*_-norm MK-SVR are listed in Table 2, too. Obviously when *p* = 1.05, 1.001, and 1.15, *ℓ*_*p*_-norm MK-SVR model performs better than SKSVR one for data1 data set, data2 data set, and data3 data set, respectively.

### 3.2. Experiment II

 Secondly, we compare the performance of *ℓ*_*p*_-norm MK-SVR with that of *ℓ*_1_-norm MK-SVR. In this experiment, the daily stock closing prices of Shanghai Stock Index in China for the period of January 2008 to December 2011 are used, and the training/validating/testing data set is generated by a one-season moving-window testing approach. Following the way done in Tay and Cao [29], three data sets, D-I to D-III, are formed. The corresponding time periods for D-I to D-III are listed in Table 3.

**Figure 3:**
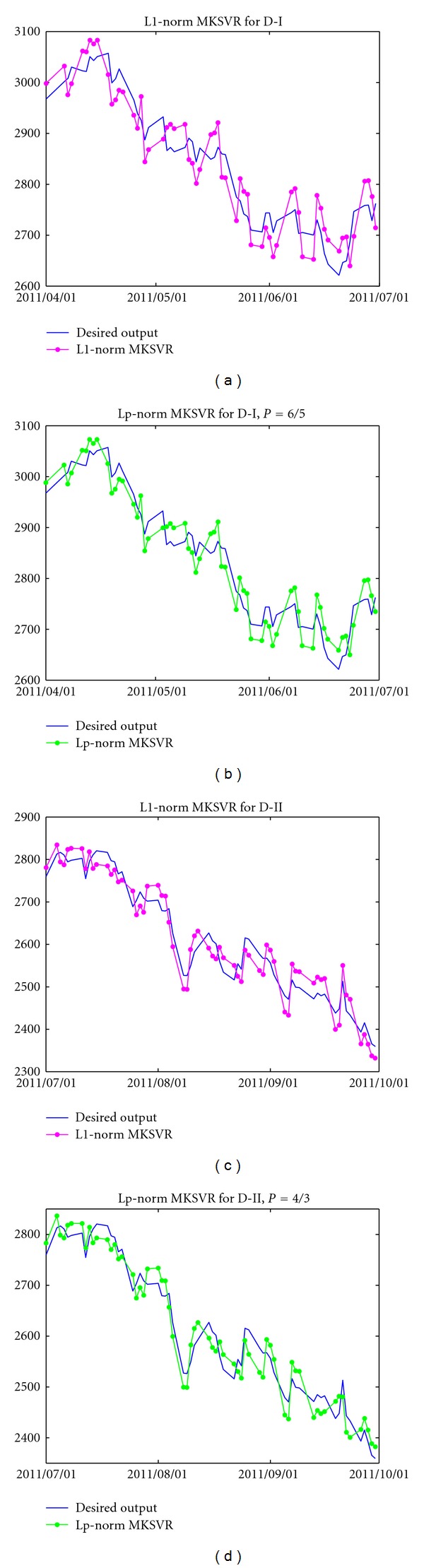
Forecasting results by *ℓ*_1_-norm MK-SVR and *ℓ*_*p*_-norm MK-SVR.

We also adopt RMSE (13) for performance comparison. For *ℓ*_1_-norm MK-SVR and *ℓ*_*p*_-norm MK-SVR training model, a kernel combining 40 different RBF kernels is considered, that is, 1/*σ* ^2^ ∈ {0.01,0.02,…, 0.09, 0.1, 0.2,…, 0.9,1, 2,…, 9,10,20,…, 100,200,300,400}. Hence, the kernel matrix is combined with a weighted sum of 40 kernel matrices,  that is, $\tilde{K}=d_{1}K_{1}+d_{2}K_{2}+\cdots +d_{40}K_{40}$ where *d*_1_ denotes the kernel weight for the first kernel matrix with 1/*σ* ^2^ = 0.01 and *d*_2_ denotes the kernel weight for the second kernel matrix with 1/*σ* ^2^ = 0.02, and so on. For the three data sets, the RMSE values obtained by *ℓ*_1_-norm MK-SVR and *ℓ*_*p*_-norm MK-SVR are listed in Table 4. Obviously when *p* = 6/5, 4/3, and 8/7, *ℓ*_*p*_-norm MK-SVR model performs better than *ℓ*_1_-norm MK-SVR one for D-I data set, D-II data set, and D-III data set, respectively.  Figure 3 shows the forecasting results for D-I and D-II by the two regression models.

**Figure 4:**
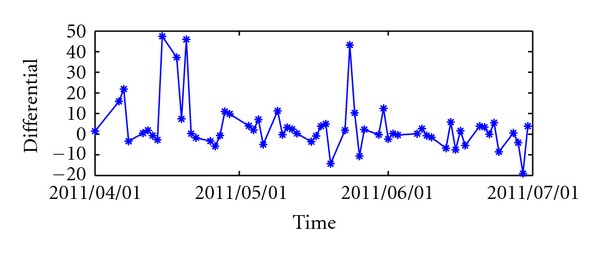
Loss differential (*ℓ*_1_-MKSVR to *ℓ*_*p*_-MKSVR) of D-I.

**Figure 5:**
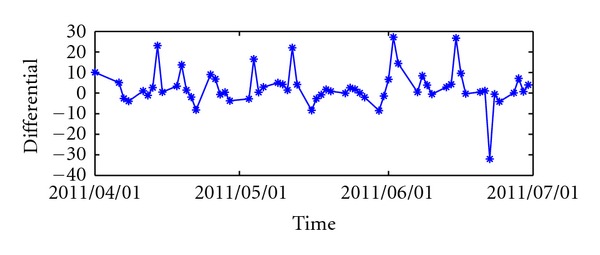
Loss differential (*ℓ*_1_-MKSVR to *ℓ*_*p*_-MKSVR) of D-II.

Furthermore, we can use a statistical test proposed by Diebold and Mariano [30] to assess the statistical significance of the forecasts by *ℓ*_*p*_-norm MK-SVR model. The loss-differential series of *ℓ*_1_-norm MK-SVR and *ℓ*_*p*_-norm MK-SVR are shown in Figures 4 and 5. According to [30], we adopt the asymptotic test $S_{1}=\overset{-}{d}/\sqrt{(2\pi {\hat{f}}_{d}(0))/T}$ as the test statistic, where *d*_*i*_ = *r*_1*i*_^2^ − *r*_2*i*_^2^ is the loss-differential series of *ℓ*_1_-norm MK-SVR and *ℓ*_*p*_-norm MK-SVR models, *r*_1_ and *r*_2_ denote the forecasting errors; $2\pi {\hat{f}}_{d}(0)$ is the weighted sum of the available sample autocovariances: $2\pi {\hat{f}}_{d}(0)=\sum_{\tau =-(T-1)}^{T-1}1*(\tau /S(T)){\hat{\gamma }}_{d}(T)$, where *T* is the sample size, ${\hat{\gamma }}_{d}(T)=(1/T)\sum_{t=|\tau |+1}^{T}(d_{t}-\overset{-}{d})(d_{t-|\vec{\tau }|}-\overset{-}{d})$, and 1∗(*τ*/*S*(*T*)) is the lag window, defined as

(14) $$\begin{array}{c} 1*\left(\frac{\tau }{S\left(T\right)}\right)=\{\begin{array}{cc} 1, & \text{if}\;\;\left|\frac{\tau }{S\left(T\right)}\right|\leq 1,\\ 0, & \text{otherwise}, \end{array} \end{array}$$

where *S*(*T*) = *k* − 1; *k* reports the number of forecasting steps ahead. 

 We denote *U*_1_ as the forecasting accuracy of *ℓ*_1_-norm MK-SVR and *U*_*p*_ as the forecasting accuracy of *ℓ*_*p*_-norm MK-SVR. Under the null hypothesis: *U*_1_ = *U*_*p*_, the test was performed at the 0.05 and 0.10 significant levels [12]. The test results are shown in the following Table 5. For the three data sets, all asymptotic tests reject *H*_0_ : *U*_1_ = *U*_*p*_. The test result shows that *ℓ*_*p*_-norm MK-SVR model indeed improves the forecasting accuracy in comparison with *ℓ*_1_-norm MK-SVR model.

We briefly mention that the superior performance of *ℓ*_*p*_-norm MK-SVR model (*p* > 1) is not surprising. When we use the sparsity-inducing norm (*p* = 1), some of the kernel weights are forced to become zero, and the corresponding kernel will be eliminated leading to some information loss. The daily stock closing prices do not carry large parts of overlapping information, and the information is discriminative. So a nonsparse kernel mixture can access more information and perform more robustly.

## 4. Summary and Prospect

In this paper, an *ℓ*_*p*_-norm MK-SVR model for stock market price forecasting is proposed. The model conceives an optimization scheme of unprecedented efficiency and provides a really efficient implementation. In an empirical evaluation, we show that *ℓ*_*p*_-norm MK-SVR can improve predictive accuracies on relevant real-world data sets. Although we focus on volatility forecasting of stock markets in this paper, our *ℓ*_*p*_-norm MK-SVR model could be applied to more general financial forecasting problems. Therefore in the future we will apply our *ℓ*_*p*_-norm MK-SVR model for other financial markets, such as exchange markets.

## Figures and Tables

**Algorithm 1:**
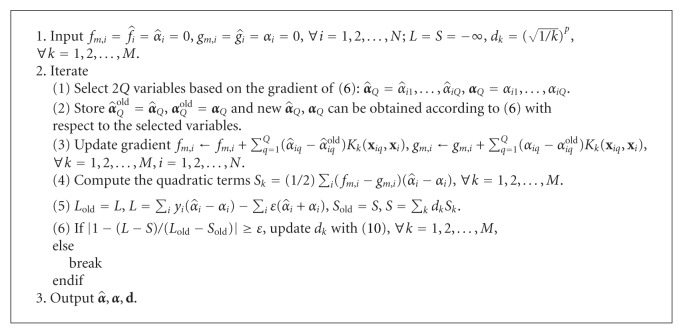


**Table 1:** The data sets for the first experiment.

Dataset	Training	Validating	Testing
data1	2003/1–2006/12	2007/1–2007/3	2007/4–2007/6
data2	2003/4–2007/3	2007/4–2007/6	2007/7–2007/9
data3	2003/7–2007/6	2007/7–2007/9	2007/10–2007/12

**Table 2:** The comparison of RMSE values between SKSVR and *ℓ*_*p*_-norm MK-SVR.

Methods	Data1	Data2	Data3
SKSVR	0.179	0.183	0.197
*ℓ*_*p*_ norm (*p* = 1.05)	**0.161**	0.177	0.186
*ℓ*_*p*_ norm (*p* = 1.001)	0.163	**0.174**	0.189
*ℓ*_*p*_ norm (*p* = 1.15)	0.166	0.179	**0.183**

**Table 3:** The data sets for the second experiment.

Dataset	Training	Validating	Testing
D-I	2008/1–2010/12	2011/1–2011/3	2011/4–2011/6
D-II	2008/4–2011/3	2011/4–2011/6	2011/7–2011/9
D-III	2008/7–2011/6	2011/7–2011/9	2011/10–2011/12

**Table 4:** The comparison of RMSE values between *ℓ*_1_-norm MK-SVR and *ℓ*_*p*_-norm MK-SVR.

Methods	D-I	D-II	D-III
*ℓ*_1_ norm	0.182	0.189	0.178
*ℓ*_*p*_ norm (*p* = 6/5)	**0.175**	0.183	0.179
*ℓ*_*p*_ norm (*p* = 4/3)	0.185	**0.181**	0.180
*ℓ*_*p*_ norm (*p* = 8/7)	0.190	0.191	**0.171**

**Table 5:** Asymptotic test.

Stock closing prices	*α* = 0.05	*α* = 0.10
D-I	*S*_1_ = 1.756,	*S*_1_ = 1.756,
	*P* value = 0.0359	*P* value = 0.0359
D-II	*S*_1_ = 1.832,	*S*_1_ = 1.832,
	*P* value = 0.0416	*P* value = 0.0416
D-III	*S*_1_ = 1.579,	*S*_1_ = 1.579,
	*P* value = 0.0258	*P* value = 0.0258

## Appendix

### *ℓ*_*p*_-Norm MK-SVR Dual Formulation

In this appendix, we detail the dual formulation of *ℓ*_*p*_-norm MK-SVR. We again consider *ℓ*_*p*_-norm MK-SVR with a general convex loss,

(A.1) minw,b 12∑k||wk||22dk+C∑i=1l(ξi+ξ^i)s.t. (〈w,K(xi)〉+b)−yi≤ε+ξi, yi−(〈w,K(xi)〉+b)≤ε+ξ^i,||d||p2≤1,d1,d2,…,dM≥0,ξ,ξ^i≥0, i=1,2,…,N.

In the following, we build the Lagrangian of (A.1). By introducing Lagrangian multipliers **α** = (*α*_1_, *α*_2_,…, *α*_*N*_)^*T*^ ∈ **R** ^*N*^, $\hat{\alpha }={\left({\hat{\alpha }}_{1},{\hat{\alpha }}_{2},\ldots ,{\hat{\alpha }}_{N}\right)}^{T}\in R^{N}$, *β* ∈ **R**_+_, and **γ** = (*γ*_1_, *γ*_2_,…, *γ*_*M*_)^*T*^∈**R** ^*M*^, the Lagrangian saddle point problem is given by

(A.2) supα,α^,η1,η2β≥0,γ≥0infw,b{12∑k||wk||22dk+C∑i=1l(ξi+ξ^i)+∑i=1Nαi(〈w,K(xi)〉+b−yi−ε−ξi)+∑i=1Nα^i(yi−〈w,K(xi)〉−b−ε−ξ^i)−γTd−η1ξ−η2ξ^+β(12||d||p2−12)}.

Set the Lagrangian's first partial derivatives with respect to **w**, *b*, *ξ*_*i*_, and ${\hat{\xi }}_{i}$, and let them be 0 to reveal the optimality conditions

(A.3) $$\begin{array}{c} w_{k}=d_{k}\sum_{i=1}N\left({\hat{\alpha }}_{i}-\alpha_{i}\right)K_{k}\left(x_{i}\right),\;k=1,2,\ldots ,M,\\ 1^{T}\left({\hat{\alpha }}_{i}-\alpha_{i}\right)=0,\;1={\left(1,1,\ldots ,1\right)}^{T},\\ C=\eta_{1i}+\alpha_{i},\\ C=\eta_{2i}+{\hat{\alpha }}_{i},\;i=1,2,\ldots ,N. \end{array}$$

Resubstituting the previous equations to the Lagrangian yields the following

(A.4) supα,α^,β≥0,γ≥0,1T(α^i−αi)=0infε,d{∑i=1Nyi(α^i−αi)−ε∑i=1N(α^i+αi)−12∑k=1Mdk·∑i=1N∑j=1N(α^i−αi)(α^j−αj)Kk(xi,xj)+β(12||d||p2−12)−γTd}

which can also be written as

(A.5) supα,α^,β≥0,γ≥0,1T(α^i−αi)=0{supε{ε∑i=1N(α^i+αi)−∑i=1Nyi(α^i−αi)}         −βsupd{1β∑k=1M(12dk∑i=1N∑j=1N(α^i−αi)(α^j−αj)             Kk(xi,xj)+γk)−12||d||p2}−12β}.

For standard support vector regression formulations, the hinge loss function can be defined as *f*(**w**; (**x**, *y*)) = max{0, |〈**w**, **x**〉−*y* | −*ε*}. This loss is also convex with a sub-gradient bounded by ||**x**||. As is known to all, the Fenchel-Legendre conjugate of a function *f* is defined as *f**(*x*) = sup_*u*_ *x* ^*T*^ *u* − *f*(*u*), and the dual form is denoted by ||·||_∗_ (the norm defined via the identity (1/2)||·||_∗_^2^ = ((1/2)||·||^2^)*. According to (A.3), (A.5), and Fenchel-Legendre conjugate of the hinge loss function, we can obtain the following dual:

(A.6)  maxα^,α,β≥0,γ≥0(yT(α^−α)−ε(α^+α))   −1β||(12dk∑i=1N∑j=1N(α^i−αi)(α^j−αj)Kk(xi,xj)+γk)k=1M||p∗2 −12β,

where **y** = (*y*_1_, *y*_2_,…, *y*_*N*_)^*T*^, **ε** = (1,1,…, 1)^*T*^, $1^{T}(\hat{\alpha }-\alpha )=0$, $0\leq \hat{\alpha },\alpha \leq C1$, and *p** = *p*/(*p* − 1).

In the following, we find $\hat{\beta }$ at optimality. Let us solve ∂*L*/∂*β* = 0 for the unbounded *β*; then we can obtain the optimal *β* as

(A.7) $$\begin{array}{c} \hat{\beta }={||{\left(\frac{1}{2}d_{k}\sum_{i=1}^{N}\sum_{j=1}^{N}\left({\hat{\alpha }}_{i}-\alpha_{i}\right)\left({\hat{\alpha }}_{j}-\alpha_{j}\right)K_{k}\left(x_{i},x_{j}\right)+\gamma_{k}\right)}_{k=1}^{M}||}_{p^{*}}. \end{array}$$

Obviously, $\hat{\beta }\geq 0$, so we can ignore the corresponding constraint from the optimization problem and plug (A.7) into (A.6). Then the following dual optimization problem for *ℓ*_*p*_-norm MK-SVR is written as  

(A.8) maxα^,α, (yT(α^−α)−ε(α^+α)) −||(12dk∑i=1N∑j=1N(α^i−αi)(α^j−αj)Kk(xi,xj)+γk)k=1M||p∗s.t. 1T(α^−α)=0,0≤α^,α≤C1,d1,d2,…,dM≥0.

For the choice of *ℓ*_*p*_ norm, *γ* = 0 holds in the optimal point so that the *γ*-term can be discarded [23]. Therefore the previous equations reduce to an optimization problem that depends on $\hat{\alpha }$ and **α** as

(A.9) maxα^,α,γ≥0 (yT(α^−α)−ε(α^+α)) −||(12dk∑i=1N∑j=1N(α^i−αi)(α^j−αj)Kk(xi,xj))k=1M||p∗s.t. 1T(α^−α)=0,0≤α^,α≤C1,d1,d2,…,dM≥0.

Now, *ℓ*_*p*_-norm MK-SVR model has been constructed.
